# ChatGPT and Microsoft Copilot for Cochlear Implant Side Selection: A Preliminary Study

**DOI:** 10.3390/audiolres15040100

**Published:** 2025-08-06

**Authors:** Daniele Portelli, Sabrina Loteta, Mariangela D’Angelo, Cosimo Galletti, Leonard Freni, Rocco Bruno, Francesco Ciodaro, Angela Alibrandi, Giuseppe Alberti

**Affiliations:** 1 Unit of Otorhinolaryngology, Department of Adult and Development Age Human Pathology “Gaetano Barresi”, University of Messina, 98125 Messina, ME, Italy; daniele.portelli09@gmail.com (D.P.); mariangeladangelo17@gmail.com (M.D.); leonardfreni@gmail.com (L.F.); brunor@unime.it (R.B.); dottfciodaro@alice.it (F.C.); galberti@unime.it (G.A.); 2 Faculty of Medicine and Surgery, Kore University of Enna, Piazza dell’Università, 94100 Enna, EN, Italy; cosimo.galletti01@unikore.it; 3 Unit of Statistical and Mathematical Sciences, Department of Economics, University of Messina, 98122 Messina, ME, Italy; aalibrandi@unime.it

**Keywords:** cochlear implants, generative artificial intelligence, artificial intelligence, sensorineural hearing loss, hearing aids, tinnitus

## Abstract

**Background/Objectives**: Artificial Intelligence (AI) is increasingly being applied in otolaryngology, including cochlear implants (CIs). This study evaluates the accuracy and completeness of ChatGPT-4 and Microsoft Copilot in determining the appropriate implantation side based on audiological and radiological data, as well as the presence of tinnitus. **Methods**: Data from 22 CI patients (11 males, 11 females; 12 right-sided, 10 left-sided implants) were used to query both AI models. Each patient’s audiometric thresholds, hearing aid benefit, tinnitus presence, and radiological findings were provided. The AI-generated responses were compared to the clinician-chosen sides. Accuracy and completeness were scored by two independent reviewers. **Results**: ChatGPT had a 50% concordance rate for right-side implantation and a 70% concordance rate for left-side implantation, while Microsoft Copilot achieved 75% and 90%, respectively. Chi-square tests showed significant associations between AI-suggested and clinician-chosen sides for both AI (*p* < 0.05). ChatGPT outperformed Microsoft Copilot in identifying radiological alterations (60% vs. 40%) and tinnitus presence (77.8% vs. 66.7%). Cronbach’s alpha was >0.70 only for ChatGPT accuracy, indicating better agreement between reviewers. **Conclusions**: Both AI models showed significant alignment with clinician decisions. Microsoft Copilot was more accurate in implantation side selection, while ChatGPT better recognized radiological alterations and tinnitus. These results highlight AI’s potential as a clinical decision support tool in CI candidacy, although further research is needed to refine its application in complex cases.

## 1. Introduction

The cochlear implant is the treatment of choice for patients with severe-to-profound bilateral sensorineural hearing loss who derive no benefit from conventional hearing aids [1,2]. This implantable device bypasses damaged cochlear hair cells to stimulate electrically the auditory nerve fibres. These devices have been proven to provide auditory perception that significantly enhances communication abilities and overall quality of life [3,4].

Cochlear implant candidacy is determined through a comprehensive clinical evaluation, including audiometric testing and detailed audiological assessment. In paediatric patients, early implantation is crucial to support language development, as the critical period for auditory system neuroplasticity can be leveraged to maximise linguistic acquisition [2]. For adults, candidacy focuses on individuals with progressive hearing loss that hinders interpersonal communication and social participation [1].

The pre-implantation process for cochlear implants is a complex multidisciplinary pathway designed to holistically and accurately assess a patient’s suitability for the procedure [5]. The multidisciplinary team involved in evaluating patients potentially eligible for cochlear implantation includes various specialists beyond the otolaryngologist and audiologist. These may include a speech therapist, radiologist, geneticist, psychiatrist or child neuropsychiatrist, cardiologist, anaesthetist, and others. The National Deaf Children’s Society and the British Cochlear Implant Group recommend that a team of at least seven specialists be involved, with the composition adaptable and expandable based on specific needs, whether for adults or children [5,6].

It is well known that several factors can influence both the candidacy and outcomes of patients undergoing cochlear implantation [7].

In recent years, artificial intelligence (AI) has emerged as a resource in medicine, aiding in diagnosis, treatment, and patient management [8,9]. In particular, generative artificial intelligence (gAI) appears to hold significant potential, offering various medical applications by providing reliable and cost-effective information [10].

AI is a branch of computer science dedicated to developing algorithms and systems capable of mimicking human cognitive functions [11]. The underlying concept is to develop systems integrated into machines with learning and adaptive capabilities, inspired by human learning models [11].

When discussing AI today, the mind immediately jumps to the latest platforms that have emerged on the web, such as chatbots like ChatGPT, Microsoft Copilot, and Google Gemini.

Chatbots represent a model of human–computer interaction and are designed to simulate human conversation, primarily through text or voice, using artificial intelligence algorithms [10,11].

These AI models are known for their ability to perform a wide range of tasks, including writing, text reviewing, translation, and adapting text to different contexts [12]. Large Language Models (LLMs) are prominent in the realm of generative AI. These systems are based on a complex network of artificial neural networks capable of mimicking the structure of the brain and handling large volumes of information [11].

LLMs have rapidly gained global traction over the past year and a half, demonstrating their potential to revolutionise the global medical field. The potential applications of LLMs in medicine primarily include medical education, scientific research, clinical practice, and the doctor–patient relationship [13,14].

By rapidly analysing large datasets and identifying complex patterns, AI enhances diagnostic accuracy, optimises treatment plans, and closely monitors clinical progress. Machine learning and deep learning algorithms, in particular, can support clinicians by interpreting images and clinical data with an accuracy comparable to that of human expertise [13,14].

In cochlear implantation, AI supports various phases, from candidate selection to post-operative mapping optimization. Advanced algorithms personalize auditory stimulation by interpreting patient responses, thereby reducing the number of sessions required to achieve optimal auditory perception [15,16,17,18].

The purpose of this study is to evaluate two widely used and freely accessible generative AI systems to assess their ability to assist and support clinicians in the pre-implantation process. For this purpose, two gAI systems, ChatGPT-4 and Microsoft Copilot, were utilised. This is a retrospective study in which specific questions with defined parameters were posed to the two gAI systems, and their responses were compared to the human decisions that had already been made. The rationale behind this study lies in the fact that, concerning clinical practice at our centre (and arguably at all centres specialising in cochlear implant surgery), the process leading up to the surgical procedure can sometimes be hindered by conditions that make it difficult to decide whether to proceed with the implantation or choose the correct side. Various factors, such as anatomical alterations detected in CT and MRI scans, the presence of comorbidities, the level of benefit from hearing aids, residual hearing, and the presence of tinnitus, can all influence the decision-making process. Patient data, including audiograms, hearing aid benefits, radiological findings of anatomical alterations, and tinnitus, were used to query the AI systems regarding cochlear implant candidacy and side selection.

## 2. Materials and Methods

### 2.1. Participants

Our retrospective, observational study was conducted at a tertiary-level audiological centre. Data from 22 adult patients who underwent cochlear implantation between January 2021 and February 2024 were used to formulate the questions posed to the two gAI systems.

Only data from adult patients over the age of 18 who received cochlear implants were included in the analysis, while data from paediatric patients were excluded.

To base this study (and thus the AI’s decision) solely on purely implantological and audiological aspects, we ensured that all the included patients had no other comorbidities or general physical conditions that would compromise the surgery.

The patients included in this pilot study were retrospectively selected from our institutional database based on the availability of complete clinical, radiological, and audiological data necessary to formulate AI queries. Although the sample size is limited, it was intentionally kept small to ensure high-quality, case-by-case data evaluation and manual review of the AI responses. The selection aimed to include representative and clinically relevant cases for a focused preliminary analysis, as detailed below.

The study was carried out following the Declaration of Helsinki. The patients provided their informed consent for the use of their clinical data in formulating AI queries. They were clearly informed that no identifying information would be included in the queries and that these were carefully constructed to ensure complete anonymity and prevent any possibility of tracing the data back to individual patients. As this is a retrospective study, patients were not directly involved in any invasive diagnostic or therapeutic tests or procedures during the study phase.

Local ethical committee approval was granted (Prot. 97-24) on 19 July 2024.

### 2.2. Generative AI

ChatGPT and Microsoft Copilot are the two generative AI-based tools used for the study.

ChatGPT (Generative Pre-Training Transformer) is a chatbot that currently operates using the OpenAI GPT-4 language architecture (https://chatgpt.com, accessed on 31 January 2025) [19]. It generates text-based (and other) responses that closely resemble those a human might provide during a conversational setting. Leveraging an extensive dataset of information, OpenAI designed ChatGPT to deliver diverse responses across a wide range of topics and cues [20]. ChatGPT, developed by OpenAI, uses machine learning to generate natural-sounding text. Its algorithm leverages language patterns and context to produce coherent and relevant responses. Widely used in fields such as customer support, education, creative and technical writing, and scientific research, ChatGPT can also adapt and learn from human feedback to enhance its response quality and interpretation skills [20].

Microsoft Copilot (https://copilot.microsoft.com, accessed on 31 January 2025), on the other hand, is a suite of AI tools designed to enhance productivity across various work environments [21]. It integrates seamlessly with office tools like text editing or mailing to help users complete tasks more efficiently. Powered by advanced AI models like GPT-4, Microsoft Copilot can interpret natural language commands and provide contextual suggestions or responses tailored to the user’s needs [22].

The AI model used by both systems to formulate the responses in this study was Open GPT-4. The queries were submitted to both AI models between September and October 2024.

### 2.3. Procedure

**Figure 1:**
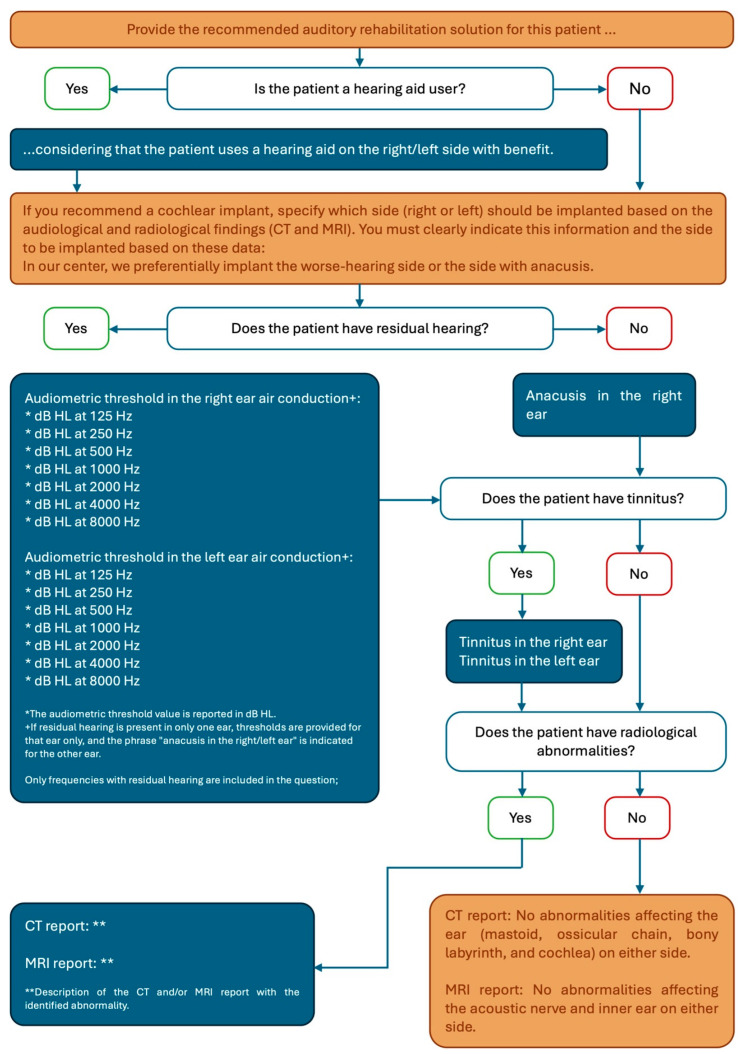
English query framework. Orange boxes represent constant sections; blue boxes represent variable sections.

The audiological and radiological data of the 22 patients were collected and catalogued by two expert audiologists. These data were used to query the two AI models: ChatGPT and Microsoft Copilot. A consistent framework was employed to formulate the questions; within this framework, variable sections were included depending on individual patient data. The questions were posed in Italian (see the Figure in the Supplementary Materials, Figure S1). Figure 1 illustrates the query framework used, showing constant (orange boxes) and variable (blue boxes) sections translated by the authors into English.

A question posed to a patient (with variable parts in italics), translated into English, is reproduced as follows for example purposes:

“Provide the auditory rehabilitation solution for this patient, *considering they are using a hearing aid with benefit on the left side.* If you recommend a cochlear implant, specify the side to be implanted (right or left) based on the audiological and radiological findings (CT and MRI). Please provide these specific details and the side to implant based on the following data:

In our centre, we preferentially implant the worse side or the side with anacusis.

*Audiometric thresholds for the right ear (air conduction):*

- *65 dB HL at 125 Hz*
- *75 dB HL at 250 Hz*
- *90 dB HL at 500 Hz*
- *90 dB HL at 1000 Hz*
- *95 dB HL at 2000 Hz*
- *105 dB HL at 4000 Hz*
- *115 dB HL at 6000 Hz*

*Audiometric thresholds for the left ear (air conduction):*

- *70 dB HL at 125 Hz*
- *80 dB HL at 250 Hz*
- *90 dB HL at 500 Hz*
- *95 dB HL at 1000 Hz*
- *95 dB HL at 2000 Hz*
- *85 dB HL at 4000 Hz*
- *90 dB HL at 6000 Hz*

*CT report: Around the cochlear turns, there are focal areas of osteorarefaction of the spongiosa.*

MRI report: No abnormalities in the vestibulocochlear nerve or the inner ear on either side.”

The formulated text was entered into the input box of both systems to query the two gAI models. The responses provided were subsequently used for statistical analysis. Finally, two additional expert audiologists independently evaluated the individual responses provided by the two gAI models for 22 patients using a Likert scale. They assigned scores ranging from 1 to 6 for response accuracy (1 being minimal accuracy and 6 being maximal accuracy) and a score from 1 to 3 for response completeness (1 being minimal completeness and 3 being maximal completeness).

For appropriateness, reviewers were required to consider four parameters when assigning a score ranging from 1 to 6 (1 = completely incorrect, 2 = more incorrect than correct, 3 = equally correct and incorrect, 4 = more correct than incorrect, 5 = almost correct, 6 = correct). This score was assigned by evaluating the appropriateness and accuracy of audiological parameters, radiological parameters, and tinnitus considerations, as well as the indicated side for implantation.

Regarding the completeness scale, responses were assessed based on three levels: incomplete, adequate, or comprehensive. An incomplete response refers to only some aspects of the query, with significant missing or incomplete parts. An adequate response addresses all aspects of the query and provides the minimum amount of required information to be considered complete. A comprehensive response covers all aspects of the query and provides additional information beyond what was requested.

### 2.4. Statistical Analysis

The study dataset includes various variables that reflect both the parameters identified by audiologists and the responses provided by the AI systems. (Refer to the table included in the Supplementary Materials for a detailed description, Table S1).

The variables of reference information identified by the audiologists include cochlear implant side, radiological abnormalities, and tinnitus.

The response variables from the AI systems are as follows: ChatGPT Side and Microsoft Copilot Side; radiological alterations considered by ChatGPT and radiological alterations considered by Microsoft Copilot; and tinnitus presence considered by ChatGPT and tinnitus presence considered by Microsoft Copilot.

The clinical data of the patients and the AI responses were compared with the specialist physician’s opinion to determine concordance or discrepancy with clinical guidelines.

For a subjective evaluation of the quality of the AI responses, two audiologist reviewers assigned accuracy and completeness scores using a Likert scale:

- Accuracy: rated from 1 to 6 to measure how precise the AI responses concerning the clinical data were;
- Completeness: rated from 1 to 3 to determine whether the AI responses included all the required elements.

The variables created for this purpose include ChatGPT Accuracy Reviewer 1, Microsoft Copilot Accuracy Reviewer 1, ChatGPT Accuracy Reviewer 2, Microsoft Copilot Accuracy Reviewer 2 and ChatGPT Completeness Reviewer 1, Microsoft Copilot Completeness Reviewer 1, ChatGPT Completeness Reviewer 2, and Microsoft Copilot Completeness Reviewer 2.

In the analysis of the AI responses, there were no cases of missing data or incomplete answers that necessitated exclusion from the evaluation.

Numerical data are expressed as means, standard deviations (SDs), and ranges (minimum and maximum), while categorical variables are presented as absolute frequencies and percentages. A non-parametric approach was adopted due to the small sample size and the non-normal distribution of the variables, verified using the Kolmogorov–Smirnov test.

The chi-square test was used to compare the following variables:

- “Cochlear implant side” vs. “ChatGPT Side” and “Cochlear implant side” vs. “Microsoft Copilot Side”;
- “Radiological Alterations” vs. “Radiological Alterations Considered by ChatGPT” and “Radiological Alterations” vs. “Radiological Alterations Considered by Microsoft Copilot”;
- “Tinnitus” vs. “Tinnitus Presence ChatGPT” and “Tinnitus” vs. “Tinnitus Presence Microsoft Copilot.”

The Cronbach’s alpha coefficient was used to verify internal consistency among the items comprising each Likert scale. The Spearman correlation test was applied to analyse possible correlations between the accuracy and completeness scores assigned by the two reviewers for each gAI.

Statistical analyses were performed using SPSS version 27.0 for Windows.

A *p*-value of less than 0.05 was considered statistically significant.

## 3. Results

Data from 22 patients were used to query the two generative AI models. Patients consisted of 11 males and 11 females, with 12 implants on the right side and 10 on the left. In 10 cases, radiological alterations were present, while 9 patients reported the presence of tinnitus. The data are summarised in Table 1.

Table 2 presents the scores for the accuracy and completeness of the responses provided by the two reviewers for each AI model, ChatGPT and Microsoft Copilot.

The chi-square test showed statistical significance when comparing the cochlear implant side with the side suggested by ChatGPT (*p*-value 0.029), as well as with the side suggested by Microsoft Copilot (*p*-value < 0.001) (Table 3).

The same test was used to compare radiological alterations with those considered by ChatGPT (*p*-value 0.002) and by Microsoft Copilot (*p*-value 0.015) (Table 4).

A significant statistical agreement was also found for tinnitus, with a comparison between tinnitus versus tinnitus presence detected by ChatGPT (*p*-value < 0.001) and tinnitus presence detected by Microsoft Copilot (*p*-value 0.001) (Table 5).

The reliability analysis showed a Cronbach’s alpha of 0.710 for the accuracy of the scores assigned by the two reviewers for ChatGPT. For the accuracy of Microsoft Copilot and the completeness of both ChatGPT and Microsoft Copilot, the Cronbach’s alpha was found to be <0.70 (Table 6).

Regarding the Spearman correlation test, a positive and significant correlation was observed only for the accuracy of the reviewers’ judgments of ChatGPT’s responses (rho 0.580, *p*-value 0.005). No other correlation was found to be statistically significant for the other variables.

## 4. Discussion

Artificial intelligence is playing an increasingly significant role in medicine, revolutionising various aspects of healthcare by enhancing diagnostic accuracy, optimising treatment plans, and improving patient outcomes [9,13]. AI-powered systems are capable of analysing vast amounts of medical data, including imaging (such as ECGs, plain radiographs, CT and MRI scans, skin images, and retinal photographs), genetic information, and electronic health records, to identify patterns and provide evidence-based recommendations [9]. In clinical practice, AI assists healthcare professionals in early disease detection, personalised treatment strategies, and workflow automation, allowing for more efficient resource allocation and reduced human error. Furthermore, AI is contributing to advancements in telemedicine and decision support systems, ultimately transforming the way healthcare is delivered and making it more accessible, precise, and patient-centred [9].

In recent years, so-called chatbots have become widely available on the web. These systems leverage AI algorithms to generate responses to user-provided queries. A chatbot consists of a core AI algorithm structure and a chat interface that allows users to interact with it [13]. The operational mechanism is quite simple, yet AI algorithms make it highly accurate in providing information. The user inputs a query in plain natural language, and the chatbot generates a textual response within seconds [13]. The conversation can continue indefinitely, with the chatbot tracking all interactions to adapt and refine its responses over time [14].

The responses provided by a chatbot can vary significantly. If the query is simple, with a clear and well-supported answer based on available web sources, or if it requires a straightforward logical or mathematical calculation, the response will almost certainly be correct. However, the situation changes when the query pertains to a question that may have multiple correct answers. When a chatbot generates an incorrect or misleading response, this phenomenon is known as “hallucination” [13,23]. Nevertheless, AI algorithms can recognise these errors and correct them automatically through self-learning mechanisms, which rely not only on information gathered by the AI but also on input provided by humans.

It is important to emphasise that the phenomenon of “hallucination” can be particularly harmful, especially when chatbots are used in the medical field. This is why ongoing research aims to evaluate the potential of generative AI to assist healthcare professionals in clinical decision-making. Whenever such systems are employed, it is imperative to verify the chatbot’s responses before considering them reliable and accurate [13].

To minimise the risk of errors in the module, we ensured a rigorous standardisation of the clinical information provided to the AI models, presenting each case in a consistent and structured format. Both ChatGPT and Microsoft Copilot were given identical queries without any prompting bias or leading instructions. Furthermore, the responses were independently evaluated by two expert reviewers, and inter-rater reliability was assessed through Cronbach’s alpha and Spearman’s correlation. While no AI tool can currently be considered entirely foolproof, these precautions aimed to maximise consistency and objectivity in the evaluation process. This study was conceived as a pilot exploration, and we acknowledge that further refinement and validation of the methodology will be necessary in larger cohorts.

Studies describing the reliability of gAI as a patient-assisting tool have been published in the oral and maxillofacial surgery, ophthalmology, and breast imaging fields [24,25,26].

In the field of otolaryngology, various AI applications are gradually emerging. For example, several studies have explored its use in oropharyngeal cancers, rhinology, and nasosinusal oncology [27,28,29,30,31].

There has been a significant increase in the scientific literature regarding the use of AI in cochlear implantation. For example, machine learning models have been used to predict the candidacy and potential outcomes [15,32,33].

The use of AI has also been applied to ear and temporal bone imaging, as well as to predict the drug release profile in cochlear implant coatings [34,35,36].

Skidmore et al. [17], in their study, used machine learning algorithms and electrophysiological measurements to predict the functional status of the cochlear nerve in individuals with cochlear implants. Their findings showed that the cochlear nerve function predicted by the model was positively and significantly correlated with speech perception performance. They concluded that the models used in their study could provide valuable support in predicting the outcomes for individuals with cochlear implants [17].

A new and intriguing aspect involves the potential use of AI within cochlear implant fitting software. The integration of AI into cochlear implants represents a significant advancement in hearing rehabilitation. Furthermore, the FOX (Fitting to Outcomes eXpert) tool (Otoconsult BV, Antwerp, Belgium), as described by Wathour et al., represents a promising approach in delivering a personalised auditory experience tailored to each individual’s unique neural profile [37,38].

In our study, we utilised two chatbots: ChatGPT and Microsoft Copilot. These chatbots are not specifically programmed to perform particular tasks but instead possess general capabilities across various domains to assist users in obtaining responses on a wide range of topics. The query can take the form of either a question or a specific command to be executed, as was the case in our study. Moreover, the query can be presented in any language and contain any type of information relevant to the response.

The rationale behind our study stems from the fact that in clinical practice, there are often various scenarios that make the decision to proceed with cochlear implantation particularly complex. These decisions are frequently discussed within a multidisciplinary team, and it is precisely in such cases that artificial intelligence—given its ability to process and integrate large amounts of data simultaneously across domains—could play a role in future clinical decision-making processes. In our study, we focused on common or relatively common cases; however, as will be discussed later in this study’s limitations, there are situations involving anatomical variations, comorbidities, syndromic deafness, ear tumours, pathologies, and specific audiological characteristics that can significantly influence the indication or the side for implantation. It is on these premises that our study may hold clinical relevance and potential applicability. Having access to a tool capable of analysing all available information in a transversal and integrated manner is undoubtedly an advantage for surgeons when faced with complex clinical cases. This is where our intention for a pilot study originated, aiming to lay the groundwork for future developments concerning the use of AI in the decision-making process for cochlear implantation.

In our case, the query consisted of a constant section applicable to all subjects under examination, along with variable sections based on individual subjects. Specifically, the data provided to the two chatbots included information regarding the use of hearing aids with benefits, audiometric thresholds for each frequency, and the possible presence of anacusis, tinnitus, or radiological abnormalities. The chatbot was therefore required to specifically provide a response regarding the side that would be implanted.

Our analysis revealed that in the case of ChatGPT, there was a 50% concordance for the right side and a 70% concordance for the left side between the AI-suggested side and the side chosen by the clinician. Similarly, for Microsoft Copilot, the concordance was 75% for the right side and 90% for the left side. The agreement in both cases was not absolute, as ChatGPT suggested bilateral implantation in five cases and did not recommend cochlear implantation in one case. A similar result was observed for Microsoft Copilot, which suggested bilateral implantation in four cases. This statistically significant result demonstrates that both AI models provided precise recommendations and suggested the implantation side in a manner closely aligned with human judgment.

A similar analysis was conducted for radiological alterations and tinnitus.

Radiological alterations indicated in the query provided to the chatbots were included in the response in 60% of cases for ChatGPT and only in 40% of cases for Microsoft Copilot. Although statistical significance was achieved, this result must be carefully interpreted, considering that some patients did not present any radiological alterations, leading the chatbots to omit such data. Additionally, it is important to acknowledge a potential bias in this aspect: the mere mention of radiological alterations in the chatbot’s response does not guarantee that these were genuinely considered when suggesting the implantation side.

Tinnitus was reported in 77.8% of ChatGPT’s responses and 66.7% of Microsoft Copilot’s responses. The same reasoning applied to radiological alterations is also relevant in this case.

Therefore, when assessing the *p*-values obtained from the chi-square test, it appears that Microsoft Copilot was more accurate in selecting the cochlear implant side, whereas ChatGPT performed better in identifying radiological alterations and tinnitus.

To date, no published studies in the scientific literature have investigated this topic using a similar methodology; as a result, it is currently not possible to carry out a meaningful comparison with findings reported by other authors.

Regarding the reliability analysis, Cronbach’s alpha was found to be greater than 0.70 only for the accuracy of ChatGPT. In contrast, for the accuracy of Microsoft Copilot and the completeness of both AI models, Cronbach’s alpha was below 0.70. Cronbach’s alpha was used to measure the consistency and agreement between the scores assigned by the two reviewers, indicating how similar their evaluations were when assessing the same content. The results showed that only for ChatGPT’s accuracy were similar scores assigned to the chatbot’s responses, as also demonstrated by a positive and significant Spearman correlation. For the other items, statistical significance was not achieved, indicating that the responses provided by the two chatbots are not subject to a single, definitive interpretation. In the study by Tepe and Emekli (2024), which compared three generative AI models, Microsoft Copilot achieved a higher score on readability scales [26]. In this study, Microsoft Copilot generated text that was more easily understandable and prioritised linguistic clarity over informational accuracy. In contrast, ChatGPT demonstrated greater accuracy in its responses. A similar result was obtained in our study, as evidenced by a Cronbach’s alpha of 0.710 solely for the accuracy of ChatGPT.

### Study Limitation

This study, of course, has several limitations: it is based on a small number of patients; the responses were reviewed by only two evaluators; the text provided to the two AI models may have influenced their responses, meaning that testing multiple queries could be ideal for fully assessing their potential; and the AI models themselves may not be sufficiently trained to handle this type of information and provide accurate recommendations.

Furthermore, our study did not include patients with cochlear malformations. It would be interesting to evaluate the AI responses in such cases or more complex clinical scenarios.

While our institution is indeed a high-volume centre, the current study was designed as a pilot investigation to explore the feasibility and potential role of generative AI models in supporting cochlear implant side decision-making. The limited sample size was deliberately chosen to facilitate a more thorough management of clinical data for each patient, as well as a careful analysis of the responses provided by the two AI tools, which were subsequently reviewed by assessors. We acknowledge that expanding the sample size in future studies would improve the statistical power, generalisability, and predictive robustness of the results.

As for future perspectives, it would be beneficial to conduct this study on paediatric patients as well, incorporating additional audiological parameters such as evoked potentials, auditory steady-state response (ASSR) thresholds, as well as the presence of associated syndromes or other comorbidities, the duration of auditory deprivation, and any other factors that could influence cochlear implant candidacy decisions. Additionally, we propose utilizing audiological functional outcome data or real ear measurements data from patients using conventional or even bone conduction hearing aids. These outcomes are typically assessed through audiological tests, particularly the Matrix sentence test, or subjective questionnaires [39,40,41,42]. Such data, especially when collected from large patient cohorts, could be leveraged to train AI models, enabling the optimal adaptation of hearing aid settings based on greater “patient-specific” precision.

The findings obtained in our study should be considered preliminary, as they represent an initial exploration of the topic and will require further validation through larger-scale studies and broader clinical applications.

## 5. Conclusions

The integration of AI into the field of cochlear implantation may represent a promising frontier, with the potential to enhance clinical decision-making and optimize patient outcomes. Our study explored the capabilities of generative AI, specifically ChatGPT and Microsoft Copilot, in providing recommendations regarding cochlear implantation. The findings imply a degree of concordance between AI-generated suggestions and clinical decisions. While generative AI can serve as a valuable tool in cochlear implant decision-making, its responses are not infallible and must be validated. The observed statistical significance in the AI models’ ability to recognise relevant audiological and radiological parameters suggests a degree of reliability, yet potential biases and the phenomenon of AI “hallucination” necessitate further investigation. Ensuring that AI algorithms accurately interpret and prioritise critical clinical information remains a key challenge.

In conclusion, AI offers exciting prospects in the realm of cochlear implantation; the ongoing validation and refinement of AI applications will be essential to harness their full potential in enhancing patient care and clinical decision-making.

## Figures and Tables

**Table 1:** Data on patients included in the study.

KERRYPNX	Frequency	Percentile (%)
Sex	22	100
Males	11	50
Females	11	50
Cochlear implant side	22	100
Right	12	54.5
Left	10	45.5
Radiological abnormalities	22	100
Yes	10	45.5
No	12	54.5
Tinnitus	22	100
Yes	9	40.9
No	13	59.1

**Table 2:** Accuracy and completeness scores were assigned by the two reviewers.

	Min	Max	Mean	SD
ChatGPT Accuracy Reviewer 1	2	6	4.77	1.478
Microsoft Copilot Accuracy Reviewer 1	4	6	5.27	0.985
ChatGPT Completeness Reviewer 1	1	3	2.27	0.883
Microsoft Copilot Completeness Reviewer 1	1	3	2.18	0.958
ChatGPT Accuracy Reviewer 2	2	6	4.82	1.468
Microsoft Copilot Accuracy Reviewer 2	2	6	5.41	1.008
ChatGPT Completeness Reviewer 2	1	3	2.45	0.596
Microsoft Copilot Completeness Reviewer 2	1	3	2.41	0.666

**Table 3:** Cochlear implant side and implant side indicated by AI.

**ChatGPT**				
		**Cochlear Implant Side**		
		**Right**	**Left**	**Total**
Chat GPT side	Right	6	1	7
		50.0%	10.0%	31.8%
	Left	2	7	9
		16.7%	70.0%	41.0%
	Bilateral	4	1	5
		33.3%	10.0%	22.7%
	No implant	0	1	1
		0.0%	10.0%	4.5%
Total		12	10	22
		100.0%	100.0%	100.0%
Pearson chi-square test				***p*** **-value**
				0.029 *
**Microsoft Copilot**				
		**Cochlear Implant Side**		
		**Right**	**Left**	**Total**
Microsoft Copilot side	Right	9	0	9
		75.0%	0.0%	40.9%
	Left	0	9	9
		0.0%	90.0%	40.9%
	Bilateral	3	1	4
		25.0%	10.0%	18.2%
Total		12	10	22
		100.0%	100.0%	100.0%
Pearson chi-square test				***p*** **-value**
				<0.001 *

* statistically significant.

**Table 4:** Presence of radiological abnormalities and AI detection.

**ChatGPT**				
		**Radiological Abnormalities**		
		**No**	**Yes**	**Total**
Radiological Alterations Considered by ChatGPT	No	12	4	16
		100.0%	40.0%	72.7%
	Yes	0	6	6
		0.0%	60.0%	27.3%
Total		12	10	22
		100.0%	100.0%	100.0%
Pearson chi-square test				***p*** **-value**
				0.002 *
**Microsoft Copilot**				
		**Radiological Abnormalities**		
		**No**	**Yes**	**Total**
Radiological Alterations Considered by Microsoft Copilot	No	12	6	18
		100.0%	60.0%	81.8%
	Yes	0	4	4
		0.0%	40.0%	18.2%
Total		12	10	22
		100.0%	100.0%	100.0%
Pearson chi-square test				***p*** **-value**
				0.015 *

* statistically significant.

**Table 5:** Presence of tinnitus and AI detection.

**ChatGPT**				
		**Tinnitus**		
		**No**	**Yes**	**Total**
Tinnitus presence ChatGPT	No	13	2	15
		100.0%	22.2%	68.2%
	Yes	0	7	7
		0.0%	77.8%	31.8%
Total		13	9	22
		100.0%	100.0%	100.0%
Pearson chi-square test				***p*** **-value**
				<0.001 *
**Microsoft Copilot**				
		**Tinnitus**		
		**No**	**Yes**	**Total**
Tinnitus presence Microsoft Copilot	No	13 100.0%	3 33.3%	16 72.7%
	Yes	0 0.0%	6 66.7%	6 27.3%
Total		13 100.0%	9 100.0%	22 100.0%
Pearson chi-square test				***p*** **-value**
				0.001 *

* statistically significant.

**Table 6:** Reliability analysis of accuracy and completeness scores.

	Cronbach’s Alpha
ChatGPT Accuracy	0.710 *
Microsoft Copilot Accuracy	0.218
ChatGPT Completeness	0.321
Microsoft Copilot Completeness	0.381

* statistically significant.

## Data Availability

The data presented in this study are available on request from the corresponding author due to the presence of information concerning the personal clinical data of the patients included in the study.

## Supplementary Materials

The following supporting information can be downloaded at: https://www.mdpi.com/article/10.3390/audiolres15040100/s1, Figure S1: Italian query framework. Orange boxes represent constant sections; blue boxes represent variable sections; Table S1: Variables list and description.

## Abbreviations

The following abbreviations are used in this manuscript:

AI	Artificial intelligence
CI	Cochlear implant
gAI	Generative artificial intelligence
LLMs	Large Language Models
FOX	Fitting to Outcomes eXpert
ASSR	Auditory steady-state response
