# Supplementary material for: ChatGPT and Microsoft Copilot for Cochlear Implant Side Selection: A Preliminary Study
Source: Audiol Res. 2025 Aug 6;15(4):100. doi: 10.3390/audiolres15040100 (PMC12383040; doi:10.3390/audiolres15040100)
Supplement: Supplementary file 1 [file audiolres-15-00100-s001.zip › Supplementary files/Table S1.docx]

**Supplementary table.** Variables list and description

| **Category** | **Variable** | **Description** |
| --- | --- | --- |
| Reference Clinical Variables  (Audiologists) | Cochlear Implant Side | Side (left or right) where the cochlear implant was applied. |
|  | Radiological Abnormalities | Presence or absence of radiological abnormalities relevant to implant candidacy. |
|  | Tinnitus | Presence or absence of tinnitus as reported by the patient. |
| AI System Responses  (ChatGPT) | ChatGPT Side | Side suggested for implantation by ChatGPT. |
|  | Radiological Alterations Considered by ChatGPT | Indicates whether ChatGPT correctly accounted radiological abnormalities in its response. |
|  | Tinnitus Presence ChatGPT | Indicates whether ChatGPT correctly accounted for the presence of tinnitus in its response. |
| AI System Responses  (Microsoft Copilot) | Microsoft Copilot Side | Side suggested for implantation by the Microsoft Copilot system. |
|  | Radiological Alterations Considered by Copilot | Indicates whether Microsoft Copilot considered radiological abnormalities in its recommendation. |
|  | Tinnitus Presence Copilot | Indicates whether Microsoft Copilot accounted for the presence of tinnitus in its response. |
| Subjective Evaluation  (Audiologists 1 for ChatGPT) | ChatGPT Accuracy  Reviewer 1 | Score (1 to 6) given by Reviewer 1 for the clinical accuracy of ChatGPT’s response. |
|  | ChatGPT Completeness Reviewer 1 | Score (1 to 3) given by Reviewer 1 for the completeness of ChatGPT’s response. |
| Subjective Evaluation  (Audiologists 2 for ChatGPT) | ChatGPT Accuracy  Reviewer 2 | Score (1 to 6) given by Reviewer 2 for the clinical accuracy of ChatGPT’s response. |
|  | ChatGPT Completeness Reviewer 2 | Score (1 to 3) given by Reviewer 2 for the completeness of ChatGPT’s response. |
| Subjective Evaluation  (Audiologists 1 for Microsoft Copilot) | Microsoft Copilot Accuracy Reviewer 1 | Score (1 to 6) given by Reviewer 1 for the clinical accuracy of Microsoft Copilot’s response. |
|  | Microsoft Copilot Completeness Reviewer 1 | Score (1 to 3) given by Reviewer 1 for the completeness of Microsoft Copilot’s response. |
| Subjective Evaluation  (Audiologists 2 for Microsoft Copilot) | Microsoft Copilot Accuracy Reviewer 2 | Score (1 to 6) given by Reviewer 2 for the clinical accuracy of Microsoft Copilot’s response. |
|  | Microsoft Copilot Completeness Reviewer 2 | Score (1 to 3) given by Reviewer 2 for the completeness of Microsoft Copilot’s response. |
