# Supplementary material for: ChatGPT and Microsoft Copilot for Cochlear Implant Side Selection: A Preliminary Study
Source: Audiol Res. 2025 Aug 6;15(4):100. doi: 10.3390/audiolres15040100 (PMC12383040; doi:10.3390/audiolres15040100)
Supplement: Supplementary file 1 [file audiolres-15-00100-s001.zip › Supplementary files/Figure S1.pdf]

Forniscimi quale è la soluzione riabilitativa uditiva per questo paziente ...

Il paziente è portatore di protesi acustiche?

Si

... considerato che è portatore a **destra/sinistra** di apparecchio acustico con beneficio.

No

Nel caso in cui consigli l'impianto cocleare l'indicazione del lato da impiantare tra destro e sinistro alla luce del quadro audiologico e radiologico (TC ed RMN). Mi devi dire specificatamente queste informazioni ed il lato da eventualmente impiantare a partire da questi dati:
Nel nostro centro impiantiamo preferenzialmente il lato peggiore o con anacusia.

Il paziente ha residui uditivi?

Si

Soglia audiometrica a **destra** per via aerea+:
* dB HL sulla frequenza 125 Hz
* dB HL sulla frequenza 250 Hz
* dB HL sulla frequenza 500 Hz
* dB HL sulla frequenza 1000 Hz
* dB HL sulla frequenza 2000 Hz
* dB HL sulla frequenza 4000 Hz
* dB HL sulla frequenza 8000 Hz

Soglia audiometrica a **sinistra** per via aerea+:
* dB HL sulla frequenza 125 Hz
* dB HL sulla frequenza 250 Hz
* dB HL sulla frequenza 500 Hz
* dB HL sulla frequenza 1000 Hz
* dB HL sulla frequenza 2000 Hz
* dB HL sulla frequenza 4000 Hz
* dB HL sulla frequenza 8000 Hz

*valore in dB HL della soglia audiometrica;
+se sono presenti residui uditivi solo in un orecchio, viene fornita la soglia solo per quell'orecchio, per l'altro viene indicata la frase "anacusia a destra/sinistra";

sono indicate solo le frequenze per cui ci sono residui uditivi, le frequenze senza residui uditvi sono state omesse dalla domanda

No

Anacusia a destra
Anacusia a sinistra

Il paziente ha acufene?

Si

Acufene a destra
Acufene a sinistra

No

Il paziente ha alterazione radiologiche?

Si

Referto TC: **

Referto RMN: **

**descrizione del referto TC e/o RMN con l'alterazione riscontrata

No

Referto TC: nessuna alterazione a carico dell'orecchio (mastoide, catena ossiculare, labirinto osseo e coclea) in entrambi i lati.

Referto RMN: nessuna alterazione a carico del nervo statoacustico e dell'orecchio interno in entrambi i
